# Genome skimming herbarium specimens for DNA barcoding and phylogenomics

**DOI:** 10.1186/s13007-018-0300-0

**Published:** 2018-06-05

**Authors:** Chun-Xia Zeng, Peter M. Hollingsworth, Jing Yang, Zheng-Shan He, Zhi-Rong Zhang, De-Zhu Li, Jun-Bo Yang

**Affiliations:** 1 Germplasm Bank of Wild Species, Kunming Institute of Botany, Chinese Academy of Sciences, Kunming, 650201 Yunnan China; 2 Royal Botanic Garden Edinburgh, 20A Inverleith Row, Edinburgh, EH3 5LR UK

**Keywords:** Degraded DNA, Herbarium specimens, Genome skimming, Plastid genome, rDNA, DNA barcoding

## Abstract

**Background:**

The world’s herbaria contain millions of specimens, collected and named by thousands of researchers, over hundreds of years. However, this treasure has remained largely inaccessible to genetic studies, because of both generally limited success of DNA extraction and the challenges associated with PCR-amplifying highly degraded DNA. In today’s next-generation sequencing world, opportunities and prospects for historical DNA have changed dramatically, as most NGS methods are actually designed for taking short fragmented DNA molecules as templates.

**Results:**

As a practical test of routine recovery of rDNA and plastid genome sequences from herbarium specimens, we sequenced 25 herbarium specimens up to 80 years old from 16 different Angiosperm families. Paired-end reads were generated, yielding successful plastid genome assemblies for 23 species and nuclear rDNAs for 24 species, respectively. These data showed that genome skimming can be used to generate genomic information from herbarium specimens as old as 80 years and using as little as 500 pg of degraded starting DNA.

**Conclusions:**

The routine plastome sequencing from herbarium specimens is feasible and cost-effective (compare with Sanger sequencing or plastome-enrichment approaches), and can be performed with limited sample destruction.

## Background

Herbaria are collections of preserved plant specimens stored for scientific study. There are approximately 3400 herbaria in the world, containing around 350 million specimens, collected over the past 400 years (http://sciweb.nybg.org/science2/indexHerbariorum.asp). These collections cover most of the world’s plant species, including many rare and endangered local endemics, and species collected from places that are currently expensive or difficult to access [1]. The recovery of DNA from this vast resource of already collected expertly-verified herbarium specimens represent a highly efficient way of building a DNA-based identification resource of the world’s plant species (DNA barcoding) and increasing knowledge of phylogenetic relationships.

The ‘unlocking’ of preserved natural history specimens for DNA barcoding/species discrimination is of particular relevance. In the first decade of DNA barcoding, it became clear that obtaining material from expertly verified is a key rate-limiting step in the construction of a global DNA reference library [2]. The millions of samples that are required for this endeavor, each needing corresponding voucher specimens and meta-data, create a strong impetus for making best-use of previously collected material.

DNA degradation in herbarium samples followed by subsequent diffusion from the sample creates challenges for DNA recovery [3]. In addition, different preservation methods can negatively affect the ability of extract, amplify and sequence DNA [4–6]. PCR amplification of historical DNA is, therefore, generally restricted to short amplicons (< 200 bp) and is further vulnerable to contamination by recent DNA and PCR products from the study species. The cumulative damage to the DNA can also cause incorrect bases to be inserted during enzymatic amplification. The main sources for these alterations are single nucleotide misincorporations [7, 8]. Above all, PCR-based Sanger sequencing by using herbarium samples to generate standard DNA barcodes can be challenging. A recent large-scale study by Kuzmina et al. 2017 [9] examined 20,816 specimens representing 5076 of 5190 vascular plant species in Canada. Kuzmina et al. found that specimen age and method of preservation had significant effects on sequence recovery for all barcode markers. However, massively-parallel short-read Next-generation sequencing (NGS) protocols have the potential to greatly increase the success of herbarium sequencing projects, as many new sequencing approaches do not rely on large, intact DNA templates and instead are well-suited for sequencing low concentrations of short (100-400 bp) fragmented molecules [3, 10].

Straub et al. [11], described how “genome skimming”, involving a shallow-pass genome sequence using NGS, could recover highly repetitive genome regions such as rDNA or organelle genomes, and yield highly useful sequence data at relatively low sequence depth, and these regions include the usual suite of DNA barcoding markers [12, 13]. The genome skimming approach using NGS has been used to recover plastid DNA and rDNA sequences from 146 herbarium specimens [14], to produce the entire nuclear genome of a 43-year-old *Arabidopsis thaliana* herbarium specimen [15], the complete plastome, the mitogenome, nuclear ribosomal DNA clusters, and partial sequences of low-copy genes from an herbarium specimen of an extinct species of *Hesperelaea* [16, 17], and the complete plastome, nuclear ribosomal DNA clusters, and partial sequences of low-copy genes from three grass herbarium specimens [18].

However, sequencing small, historical specimens may be especially challenging if a specimens is unique, or nearly so, with no alternative specimens available for study should the first specimen fail. Methods used to extract and prepare DNA for sequencing must both be more or less guaranteed to work, and, in many cases, allow for preservation of DNA for future study [19]. In recent studies that report successfully sequencing of historical specimens from 1 ng to 1 μg of input DNA (for example, up to 1 μg in Bakker et al. [14]; ∽ 600 ng in Staats et al. [15]; 33 ng in Zadane et al. [17]; 8.25–537 ng in Kanda et al. [20]; 5.8–200 ng in Blaimer et al. [21]; less than 10 ng in Besnard et al. [18]; 1–10 ng in Sproul and Maddison [19]). But a number of studies also report abandoning a subset of specimens for which too little input DNA was available (i.e. below 10 ng in Kanda et al. [20]; below 5 ng in Blaimer et al. [21]). To better understand ideal approaches of sample preparation for specimens with minimal DNA, we intentionally limited DNA input to 500 pg per specimen.

In this paper we provide a further practical test of the genome skimming methodology applied to herbarium specimens. As part of the China Barcode of Life project, and our wider phylogenomic studies, our aim was to assess whether the success reported in these early genome skimming studies could be repeated in other laboratories.

We evaluated the success and failure rates of rDNA and plastid genome sequencing from genome skims of 25 different species from herbarium specimens, and explored the impacts of parameters such as amount of input DNA and PCR cycle numbers.

## Methods

### Specimen sampling

25 herbarium specimens were selected from 16 Angiosperm families covering 22 genera, with specimen ages up to 80 years old. All 25 species were taken from the specimens housed in the Herbarium of the Institute of Botany, Chinese Academy of Sciences (KUN). The samples were selected to represent the major clades of APG III system (Table 1).

**Table 1:** List of the specimen materials, DNA yields used in our study

Sample ID	Species	Family	Collection	Age	ng/ul	Volume (ul)	DNA yield (ng)
01	*Manglietia fordiana*	Magnoliaceae	19780402	39	0.894	36	32.184
02	*Manglietia fordiana*	Magnoliaceae	19541027	63	2.35	37	86.95
03	*Schisandra henryi*	Schisandraceae	19821108	35	1.87	33	61.71
04	*Schisandra henryi*	Schisandraceae	19840528	33	0.909	33	29.997
05	*Phoebe neurantha*	Lauraceae	1938	79	0.507	36	18.252
06	*Cinnamomum bodinieri*	Lauraceae	1960	57	2.26	36	81.36
08	*Holboellia latifolia*	Lardizabalaceae	1982	35	1.29	34	43.86
09	*Chloranthus erectus*	Chloranthaceae	1973	44	4.18	36	150.48
10	*Sarcandra glabra*	Chloranthaceae	1988	29	4.35	31.5	137.025
11	*Meconopsis racemosa*	Papaveraceae	1976	41	4.35	22	95.7
12	*Macleaya microcarpa*	Papaveraceae	1986	31	1.97	35.5	69.935
13	*Hodgsonia macrocarpa*	Cucurbitaceae	1982	35	2.18	34	74.12
14	*Malus yunnanensis*	Rosaceae	1939	78	0.834	35	29.19
15	*Elaeagnus loureirii*	Elaeagnaceae	1993	24	9.75	34	331.5
16	*Rhododendron rex* subsp. *fictolacteum*	Ericaceae	1979	38	8.15	20.5	167.075
17	*Swertia bimaculata*	Gentianaceae	19840823	33	1.67	35	58.45
18	*Primula sinopurpurea*	Primulaceae	19400907	77	0.974	32	31.168
19	*Paederia scandens*	Araceae	19550331	62	0.344	34	11.696
20	*Colocasia esculenta*	Araceae	19741001	43	1.46	36	52.56
21	*Pholidota chinensis*	Orchidaceae	1959	58	0.107	34	3.638
22	*Otochilus porrectus*	Orchidaceae	1990	27	0.344	35	12.04
23	*Indosasa sinica*	Poaceae	2007	10	1.65	35	57.75
24	*Camellia gymnogyna*	Theaceae	19340617	83	0.417	36	15.012
25	*Camellia sinensis* var. *assamica*	Theaceae	2002	15	4.03	23	92.69
26	*Panicum incomtum*	Poaceae	20001017	17	1.63	36	58.68

All vouchers are deposited in the herbarium of the Kunming Institute of Botany (KUN)

### DNA extraction

Approximately 1 cm^2^ sections of leaf or 20 mg of leaf tissue were used for each DNA extraction. Genomic DNA was extracted using Tiangen DNAsecure Plant Kit (DP320). Yield and integrity (size distribution) of genomic DNA extracts were quantified by fluorometric quantification on the Qubit (Invitrogen, Carlsbad, California, USA) using the dsDNA HS kit, as well as by visual assessment on a 1% agarose gel.

### Library preparation

All samples were subsequently built into blunt-end DNA libraries in the laboratories using the NEBNext Ultra II DNA library Prep kit for Illumina (New England BIolabs) which has been optimized for as little as 5 ng starting DNA and Illumina-specific adapters [22]. The library protocol was performed as per the manufacturer’s instructions with four modifications: (i) 500 pg of input DNA was selected to accommodate low starting DNA quantities, (ii) DNA was not fragmented by sonication because the DNA was highly degraded; (iii) The NEBNext library was generated without any size selection; (iv) DNA libraries were then amplified in an indexing PCR, which barcoded each library and discriminated each sample. Five PCR cycles was suggested by the manufacturer’s instruction for 5 ng of input DNA. As only 500 pg of starting DNA was used, we tested use of increasing numbers of PCR cycles (namely × 6, × 8, × 10, × 12, × 14 PCR cycles). Concentration and size profiles of the final indexed libraries (125 libraries, representing 25 specimens at 5 different numbers of PCR cycles) were assessed on a Bioanalyzer 2100 using a high sensitivity DNA chip.

### Library pooling

The final indexed libraries were then pooled (33 or 34 samples per lane) in equimolar ratios and sequenced on three lanes on an Illumina XTen sequencing system (Illumina Inc.) using paired and chemistry at the Cloud health Medical Group Ltd.

### Analyses

Successfully sequenced samples were assembled into chloroplast genomes and nuclear rDNAs. Here the rDNAs comprise the complete sequence of 26S, 18S, and 5.8S and internal transcribed spacers (ITS1 and ITS2). We did not assemble the internal gene spacer (IGS) because of the complexity of this region which is rich in duplications and inversions.

The raw sequence reads were filtered for primer/adaptor sequences and low-quality reads with the NGS QC Toolkit [23]. The cut-off value for percentage of read length was 80, and that for PHRED quality score was 30. Then the filtered high-quality pair-end reads were assembled into contigs with Spades 3.0 [24]. Next, we identified highly similar genome sequences using the Basic Local Alignment Search Tool (BLAST: http://blast.ncbi.nlm.gov/). The procedures and parameters for setting the sequence quality control, de novo assembly, and blast search were followed as in Yang et al. [25]. Next, we determined the proper orders of the aligned contigs using the highly similar genome sequences identified in the BLAST search as references. At this point, the target contigs were assembled into complete plastid genomes and nuclear rDNAs.

Annotation of the plastomes was performed using the plastid genome annotation package DOGMA [26] (http://dogma.ccbb.utexas.edu/). Start and stop codons of protein-coding genes, as well as intron/exon positions, were manually adjusted. The online tRNAscan-SE service [27] was used to further determine tRNA genes. The final complete plastomes and rDNAs were deposited into GenBank (Accession numbers: MH394344-MH394431; MH270450-MH270494).

Fungi or other plants may be co-isolated during the DNA extraction process resulting in DNA contamination [1]. This is particularly important where starting DNA concentrations are extremely low. We thus sub-sampled our data to check for contamination. To check for contamination in the plastid DNA sequences, for each species we extracted its *rbcL* sequence and blasted it against GenBank to check that it grouped with related species. BLAST1 (implemented in the BLAST program, version 2.2.17) was used to search the reference database for each query sequence with an E value < 1 × 10^−5^. Likewise, to check for plant and fungal contamination in the rDNA sequences, we took the final assembled ITS sequences (or partial ITS sequences where complete ITS was not recovered) and blasted the sequences against the NCBI database to check that it grouped with related species.

## Results

**Fig. 1:**
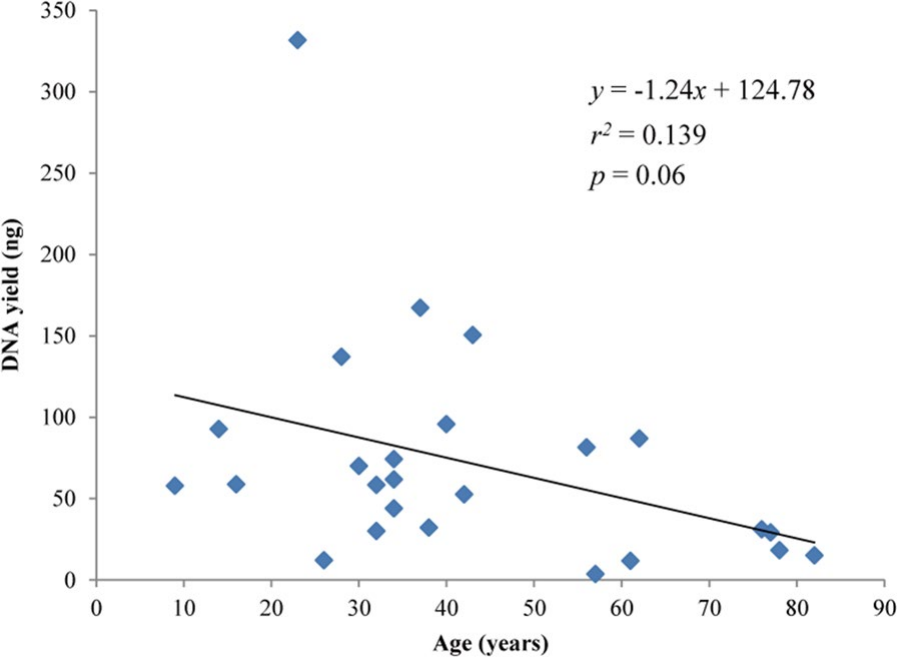
DNA yield against specimen age

All 25 species yielded amounts of DNA suitable for library preparation and further processing. Total yields varied between 3 ng and 400 ng from on average 20 mg of dried leaf tissue, usually the equivalent of 1 cm^2^ of leaf tissue (Table 1). We found a negative correlation between specimen age and DNA yield (Fig. 1).

We successfully enriched and sequenced DNA libraries constructed from herbarium material. Despite only 500 pg of input DNA, good quality libraries were produced from 100 of 125 samples (25 species, with × 8, × 10, × 12, × 14 PCR cycles). The concentration of the final indexed libraries based on six PCR cycles per species was too low to be further sequenced. Between 15,877,478 and 44,724,436 high-quality paired-end reads were produced, with the total number of bases ranging from 2,381,621,700 bp (2.38 giga base pairs, Gbp) to 6,708,665,400 bp (6.71 Gbp) (Table 2). These were then assembled into contigs, and using a blast search into plastid genomes and rDNA arrays.

**Table 2:** Assembly statistics of plastid genome for all specimens used in this study

Sample ID	PCR cycles	Species	Family	Total sequences	Raw data (gb)	#contigs	Total assembly length (bp)	Completed	GenBank accession number
01D	×8	*Manglietia fordiana*	Magnoliaceae	22404632	3.36	9	158993	1059 bp gap	MH394393
01E	×10	*Manglietia fordiana*	Magnoliaceae	25869654	3.88	32	159759	349 bp gap	MH394394
01A	×12	*Manglietia fordiana*	Magnoliaceae	35201972	5.28	14	158241	1840 bp gap	MH394391
01B	×14	*Manglietia fordiana*	Magnoliaceae	30007234	4.5	14	158221	1840 bp gap	MH394392
02D	×8	*Manglietia fordiana*	Magnoliaceae	22829038	3.42	8	161497	1040 bp gap	MH394397
02E	×10	*Manglietia fordiana*	Magnoliaceae	32497068	4.87	21	160113	Y	MH394398
02A	×12	*Manglietia fordiana*	Magnoliaceae	29637182	4.45	12	158315	1802 bp gap	MH394395
02B	×14	*Manglietia fordiana*	Magnoliaceae	31089730	4.66	22	160113	Y	MH394396
03D	×8	*Schisandra henryi*	Schisandraceae	29691984	4.45	5	145963	94 bp gap	MH394365
03E	×10	*Schisandra henryi*	Schisandraceae	25141160	3.77	4	145616	54 bp gap	MH394366
03A	×12	*Schisandra henryi*	Schisandraceae	32511344	4.88	11	146031	18 bp gap	MH394363
03B	×14	*Schisandra henryi*	Schisandraceae	29856636	4.48	9	145993	63 bp gap	MH394364
04D	×8	*Schisandra henryi*	Schisandraceae	24039822	3.61	4	146212	53 bp gap	MH394369
04E	×10	*Schisandra henryi*	Schisandraceae	23870902	3.58	4	146243	53 bp gap	MH394370
04A	×12	*Schisandra henryi*	Schisandraceae	33190158	4.98	15	146218	63 bp gap	MH394367
04B	×14	*Schisandra henryi*	Schisandraceae	30498044	4.57	6	145893	45 bp gap	MH394368
05D	×8	*Phoebe neurantha*	Lauraceae	29040850	4.36	11	152782	Y	MH394354
05E	×10	*Phoebe neurantha*	Lauraceae	27831254	4.17	15	152782	Y	MH394355
05A	×12	*Phoebe neurantha*	Lauraceae	44724436	6.71	17	152781	1 bp gap	MH394352
05B	×14	*Phoebe neurantha*	Lauraceae	35264634	5.29	13	152781	1 bp gap	MH394353
06D	×8	*Cinnamomum bodinieri*	Lauraceae	30188820	4.53	9	152778	Y	MH394417
06E	×10	*Cinnamomum bodinieri*	Lauraceae	32065328	4.81	13	152719	Y	MH394418
06A	×12	*Cinnamomum bodinieri*	Lauraceae	24488292	3.67	7	152719	Y	MH394415
06B	×14	*Cinnamomum bodinieri*	Lauraceae	35035602	5.26	11	152719	Y	MH394416
08D	×8	*Holboellia latifolia*	Lardizabalaceae	26229946	3.93	5	157817	Y	MH394377
08E	×10	*Holboellia latifolia*	Lardizabalaceae	28273022	4.24	9	157818	Y	MH394378
08A	×12	*Holboellia latifolia*	Lardizabalaceae	33873136	5.08	13	157614	204 bp gap	MH394375
08B	×14	*Holboellia latifolia*	Lardizabalaceae	34021360	5.1	10	157818	Y	MH394376
09D	×8	*Chloranthus erectus*	Chloranthaceae	21843512	3.28	4	157812	43 bp gap	MH394413
09E	×10	*Chloranthus erectus*	Chloranthaceae	18044364	2.71	5	157812	47 bp gap	MH394414
09A	×12	*Chloranthus erectus*	Chloranthaceae	30022162	4.5	13	157852	Y	MH394411
09B	×14	*Chloranthus erectus*	Chloranthaceae	28656686	4.3	11	157852	Y	MH394412
10D	×8	*Sarcandra glabra*	Chloranthaceae	18893508	2.83	5	158733	119 bp gap	MH394361
10E	×10	*Sarcandra glabra*	Chloranthaceae	20662770	3.1	7	159007	22 bp gap	MH394362
10A	×12	*Sarcandra glabra*	Chloranthaceae	27510166	4.13	9	158900	Y	MH394360
10B	×14	*Sarcandra glabra*	Chloranthaceae	29545206	4.43	9	158900	Y	MH394431
11D	×8	*Meconopsis racemosa*	Papaveraceae	24351884	3.65	5	153762	Y	MH394401
11E	×10	*Meconopsis racemosa*	Papaveraceae	29160582	4.37	5	153762	Y	MH394402
11A	×12	*Meconopsis racemosa*	Papaveraceae	33763340	5.06	6	153763	Y	MH394399
11B	×14	*Meconopsis racemosa*	Papaveraceae	35990358	5.4	4	153728	1 bp gap	MH394400
12D	×8	*Macleaya microcarpa*	Papaveraceae	26265548	3.94	11	161064	48 bp gap	MH394385
12E	×10	*Macleaya microcarpa*	Papaveraceae	25100372	3.77	11	161064	48 bp gap	MH394386
12A	×12	*Macleaya microcarpa*	Papaveraceae	29491952	4.42	13	161118	Y	MH394383
12B	×14	*Macleaya microcarpa*	Papaveraceae	28462338	4.27	12	161110	2 bp gap	MH394384
13D	×8	*Hodgsonia macrocarpa*	Cucurbitaceae	26886870	4.03	26	155027	1300 bp gap	MH394428
13E	×10	*Hodgsonia macrocarpa*	Cucurbitaceae	34179418	5.13	16	154855	1298 bp gap	MH394429
13A	×12	*Hodgsonia macrocarpa*	Cucurbitaceae	37182144	5.58	18	156015	20 bp gap	MH394426
13B	×14	*Hodgsonia macrocarpa*	Cucurbitaceae	36782268	5.52	17	156146	Y	MH394427
14D	×8	*Malus yunnanensis*	Rosaceae	22107718	3.32	16	158955	820 bp gap	MH394389
14E	×10	*Malus yunnanensis*	Rosaceae	25720160	3.86	5	160071	Y	MH394390
14A	×12	*Malus yunnanensis*	Rosaceae	37501036	5.63	5	160067	Y	MH394387
14B	×14	*Malus yunnanensis*	Rosaceae	33776058	5.07	5	160068	Y	MH394388
15D	×8	*Elaeagnus loureirii*	Elaeagnaceae	15195822	2.28	5	152196	8 bp gap	MH394424
15E	×10	Elaeagnus *loureirii*	Elaeagnaceae	16862680	2.53	5	152196	8 bp gap	MH394425
15A	×12	*Elaeagnus loureirii*	Elaeagnaceae	21511050	3.23	4	152199	5 bp gap	MH394422
15B	×14	*Elaeagnus loureirii*	Elaeagnaceae	20556860	3.08	6	152199	5 bp gap	MH394423
16D	×8	*Rhododendron rex* subsp. *fictolacteum*	Ericaceae	23623070	3.54				
16E	×10	*Rhododendron rex* subsp. *fictolacteum*	Ericaceae	28092596	4.21				
16A	×12	*Rhododendron rex* subsp. *fictolacteum*	Ericaceae	31352560	4.7				
16B	×14	*Rhododendron rex* subsp. *fictolacteum*	Ericaceae	30525730	4.58				
17D	×8	*Swertia bimaculata*	Gentianaceae	18303136	2.77	53	152808	266 bp gap	MH394373
17E	×10	*Swertia bimaculata*	Gentianaceae	16559554	2.48	41	153443	406 bp gap	MH394374
17A	×12	*Swertia bimaculata*	Gentianaceae	15877478	2.38	30	143977	9947 bp gap	MH394371
17B	×14	*Swertia bimaculata*	Gentianaceae	18448302	2.77	48	153602	341 bp gap	MH394372
18D	×8	*Primula sinopurpurea*	Primulaceae	22890598	3.43	5	151945	50 bp gap	MH394358
18E	×10	*Primula sinopurpurea*	Primulaceae	26618684	3.99	5	151945	50 bp gap	MH394359
18A	×12	*Primula sinopurpurea*	Primulaceae	24107472	3.62	3	151945	50 bp gap	MH394356
18B	×14	*Primula sinopurpurea*	Primulaceae	25834066	3.88	3	151945	50 bp gap	MH394357
19D	×8	*Paederia scandens*	Araceae	25307356	3.8	15	162267	247 bp gap	MH394346
19E	×10	*Paederia scandens*	Araceae	24658068	3.7	7	162268	247 bp gap	MH394347
19A	×12	*Paederia scandens*	Araceae	23850180	3.58	8	162282	253 bp gap	MH394344
19B	×14	*Paederia scandens*	Araceae	24064764	3.61	10	162139	253 bp gap	MH394345
20D	×8	*Colocasia esculenta*	Araceae	29284270	4.39	4	162350	155 bp gap	MH394430
20E	×10	*Colocasia esculenta*	Araceae	25045978	3.77	5	162350	155 bp gap	MH394421
20A	×12	*Colocasia esculenta*	Araceae	23560322	3.53	6	162414	155 bp gap	MH394419
20B	×14	*Colocasia esculenta*	Araceae	24533656	3.68	4	162414	155 bp gap	MH394420
21D	×8	*Pholidota chinensis*	Orchidaceae	21688990	3.25				
21E	×10	*Pholidota chinensis*	Orchidaceae	20880950	3.13				
21A	×12	*Pholidota chinensis*	Orchidaceae	23548018	3.53				
21B	×14	*Pholidota chinensis*	Orchidaceae	27148284	4.07				
22D	×8	*Otochilus porrectus*	Orchidaceae	15550512	2.33				
22E	×10	*Otochilus porrectus*	Orchidaceae	22638772	3.4				
22A	×12	*Otochilus porrectus*	Orchidaceae	21572196	3.23				
22B	×14	*Otochilus porrectus*	Orchidaceae	28960858	4.34				
23D	×8	*Indosasa sinica*	Gramineae	18793020	2.82	6	139848	18 bp gap	MH394381
23E	×10	*Indosasa sinica*	Gramineae	17903432	2.69	10	139740	Y	MH394382
23A	×12	*Indosasa sinica*	Gramineae	19106404	2.87	9	139740	Y	MH394379
23B	×14	*Indosasa sinica*	Gramineae	19668682	2.95	8	139740	Y	MH394380
24D	×8	*Camellia gymnogyna*	Theaceae	17176632	2.58	4	156402	Y	MH394405
24E	×10	*Camellia gymnogyna*	Theaceae	24532196	3.68	7	156590	Y	MH394406
24A	×12	*Camellia gymnogyna*	Theaceae	26478224	3.97	4	156590	Y	MH394403
24B	×14	*Camellia gymnogyna*	Theaceae	29768770	4.47	4	156590	Y	MH394404
25D	×8	*Camellia sinensis* var. *assamica*	Theaceae	23291572	3.49	4	157028	Y	MH394409
25E	×10	*Camellia sinensis* var. *assamica*	Theaceae	18698814	2.8	5	157028	Y	MH394410
25A	×12	*Camellia sinensis* var. *assamica*	Theaceae	21788776	3.27	4	157029	Y	MH394407
25B	×14	*Camellia sinensis* var. *assamica*	Theaceae	26155342	3.92	8	157028	Y	MH394408
26D	×8	*Panicum incomtum*	Gramineae	16865102	2.53	61	139986	Y	MH394350
26E	×10	*Panicum incomtum*	Gramineae	20465942	3.07	21	139999	Y	MH394351
26A	×12	*Panicum incomtum*	Gramineae	20004364	3	18	139999	Y	MH394348
26B	×14	*Panicum incomtum*	Gramineae	20672642	3.1	17	139999	Y	MH394349

After de novo assembly, two species (*Otochilus porrectus* and *Pholidota chinensis*) generated poor plastid assemblies, with the longest contigs being 6705 bp with 2 × coverage and 1325 bp with 3 × coverage respectively. The other 23 species yielded useful plastid assemblies drawn from 3 to 61 contigs assembled into plastid genomes with depths ranged from 459 × to 2176 ×. Of these 23 species, 14 were assembled into complete plastid genomes. Eight species were assembled into nearly complete plastid genomes, but with gaps ranged from 5 to 349 bp (Table 2). However, although *Rhododendron rex* subsp. *fictolacteum* yielded useful plastid assemblies, many gaps were detected among contigs when the species *Vaccinium macrocarpon* was used as reference data.

For the nuclear rDNAs, 21 species gave ribosomal DNA sequences assemblies > 4.3 kb drawn from 1 to 2 contigs with sequencing depths ranging from 3 × to 567 × (no nrDNA sequences could be assembled for *Phodidota chinensis, Paederia scandens, Otochilus porrectus*, and *Camellia gymnogyna*) (Table 3). Of these 21 species, 18 resulted in assembled nrDNAs consisting of partial sequences of 18S and 26S, along with the complete sequence of 5.8S and the internal transcribed spacers ITS1 and ITS2. However, 3 species (2 samples of *Manglietia fordiana* (Sample ID 01 and 02), *Phoebe neurantha* (Sample ID 05), were difficult to assemble, resulting in only partial recovery of 5.8S and the internal transcribed spacers ITS1 and ITS2.

**Table 3:** Assembly statistics of rDNAs for all specimens used in this study

Sample ID	PCR Cycles	Species	Family	#contigs	Total assembly length (bp)	(mean) Coverage (×)	Reference genome	GenBank accession number
01A	×12	*Manglietia fordiana*	Magnoliaceae	2	10343	406	KJ414477_*Chrysobalanus icaco*	MH270473
02A	×12	*Manglietia fordiana*	Magnoliaceae	2	8637	67		MH270474
03A	×12	*Schisandra henryi*	Schisandraceae	1	15487	47		MH270475
04A	×12	*Schisandra henryi*	Schisandraceae	1	10747	78		MH270476
05A	×12	*Phoebe neurantha*	Lauraceae	2	7516	19		MH270477
06A	×12	*Cinnamomum bodinieri*	Lauraceae	1	10926	32		MH270478
08A	×12	*Holboellia latifolia*	Lardizabalaceae	1	9298	160		MH270479
09A	×12	*Chloranthus erectus*	Chloranthaceae	1	9094	54		MH270480
10A	×12	*Sarcandra glabra*	Chloranthaceae	1	9062	51		MH270481
11A	×12	*Meconopsis racemosa*	Papaveraceae	1	7577	60		MH270482
12A	×12	*Macleaya microcarpa*	Papaveraceae	1	12587	458		MH270483
13A	×12	*Hodgsonia macrocarpa*	Cucurbitaceae	1	10172	567		MH270484
14A	×12	*Malus yunnanensis*	Rosaceae	1	5953	249		MH270485
15A	×12	*Elaeagnus loureirii*	Elaeagnaceae	1	7901	428		MH270486
16A	×12	*Rhododendron rex* subsp. *fictolacteum*	Ericaceae	1	6825	380		MH270487
17A	×12	*Swertia bimaculata*	Gentianaceae	1	9644	48		MH270488
18A	×12	*Primula sinopurpurea*	Primulaceae	1	5539	15		MH270489
19A	×12	*Paederia scandens*	Araceae					
20A	×12	*Colocasia esculenta*	Araceae	1	4399	5		MH270490
21A	×12	*Pholidota chinensis*	Orchidaceae	–	–	–		–
22A	×12	*Otochilus porrectus*	Orchidaceae					
23A	×12	*Indosasa sinica*	Gramineae	1	17306	93		MH270491
24A	×12	*Camellia gymnogyna*	Theaceae					
25A	×12	*Camellia sinensis* var. *assamica*	Theaceae	1	11212	46		MH270493
26A	×12	*Panicum incomtum*	Gramineae	1	8446	74		MH270494

To check the quality of the plastid sequences, all gene regions were translated. No stop codons that would be indicative of sequencing errors were detected within the assembled contigs. We then extracted about 1400 bp of *rbcL* sequence from 23 of the samples to check for contamination (for *Rhododendron rex* subsp. *fictolacteum* (Sample ID 16), the plastid genome was not assembled successfully but we could nevertheless extract the *rbcL* sequence from the plastid contigs). These *rbcL* sequences were subjected to a blast search against the NCBI database. The *rbcL* sequences contained no insertions or deletions and matched the correct genus or family in each case (Table 4). Likewise, we blasted the final assembled rDNA ITS sequences (or partial ITS sequences) from 24 samples against the NCBI database. In all cases, the closest match to the sequence was from the family of the sequenced sample. No matches with fungi were detected (Table 5).

**Table 4:** BLAST results with extracted *rbcL* sequence against GenBank

Query Information					BLAST results			
Query_Sample ID	Query_Species (Family)	PCR cycles	Gene name	Length (bp)	Reference_Species_Accession number (Family)	Query coverage (%)	Identities (%)	Identify level
01A	*Manglietia fordiana* (Magnoliaceae)	12	rbcL	1428	*Magnolia cathcartii_*JX280392.1 (Magnoliaceae)	100	99	Family
					*Magnolia biondii*_KY085894.1 (Magnoliaceae)	100	99	
					*Michelia odora*_JX280398.1 (Magnoliaceae)	100	99	
					*Manglietia fordiana*_L12658.1 (Magnoliaceae)	98	100	
02A	*Manglietia fordiana* (Magnoliaceae)	12	rbcL	1428	*Magnolia cathcartii_*JX280392.1 (Magnoliaceae)	100	99	Family
					*Magnolia biondii*_KY085894.1 (Magnoliaceae)	100	99	
					Michelia odora_JX280398.1 (Magnoliaceae)	100	99	
					*Manglietia fordiana*_L12658.1 (Magnoliaceae)	98	100	
03A	*Schisandra henryi* (Schisandraceae)	12	rbcL	1428	*Schisandra chinensis_*KY111264.1 (Schisandraceae)	100	99	Genus
					*Schisandra chinensis_*KU362793.1 (Schisandraceae)	100	99	
					*Schisandra sphenanthera_*L12665.2 (Schisandraceae)	98	99	
04A	*Schisandra henryi* (Schisandraceae)	12	rbcL	1428	*Schisandra chinensis_*KY111264.1 (Schisandraceae)	100	99	Genus
					*Schisandra chinensis_*KU362793.1 (Schisandraceae)	100	99	
					*Schisandra sphenanthera_*L12665.2 (Schisandraceae)	98	99	
05A	*Phoebe neurantha* (Lauraceae)	12	rbcL	1428	*Phoebe omeiensis_*KX437772.1 (Lauraceae)	100	99	Family
					*Persea Americana_*KX437771.1 (Lauraceae)	100	99	
					*Persea* sp. *_*JF966606.1 (Lauraceae)	100	99	
06A	*Cinnamomum bodinieri* (Lauraceae)	12	rbcL	1428	*Phoebe bournei_*KY346512.1 (Lauraceae)	100	99	Family
					*Phoebe chekiangensis*_KY346511.1 (Lauraceae)	100	99	
					*Phoebe sheareri_*KX437773.1 (Lauraceae)	100	99	
					*Cinnamomum verum*_KY635878.1 (Lauraceae)	100	99	
08A	*Holboellia latifolia* (Lardizabalaceae)	12	rbcL	1428	*Akebia quinata_*KX611091.1 (Lardizabalaceae)	100	99	Family
					*Stauntonia hexaphylla*_L37922.2 (Lardizabalaceae)	99	99	
					*Akebia trifoliate*_KU204898.1 (Lardizabalaceae)	100	99	
					*Holboellia latifolia_*L37918.2 (Lardizabalaceae)	99	99	
09A	*Chloranthus erectus* (Chloranthaceae)	12	rbcL	1428	*Chloranthus spicatus_*EF380352.1 (Chloranthaceae)	100	100	Genus
					*Chloranthus japonicas_*KP256024.1 (Chloranthaceae)	100	99	
					*Chloranthus spicatus*_AY236835.1 (Chloranthaceae)	98	99	
					*Chloranthus erectus_*AY236834.1 (Chloranthaceae)	98	99	
10A	*Sarcandra glabra* (Chloranthaceae)	12	rbcL	1428	*Chloranthus spicatus_*EF380352.1 (Chloranthaceae)	100	99	Family
					*Chloranthus japonicas_*KP256024.1 (Chloranthaceae)	100	98	
					*Chloranthus nervosus*_AY236841.1 (Chloranthaceae)	97	98	
					*Sarcandra glabra_*HQ336522.1 (Chloranthaceae)	89	100	
11A	*Meconopsis racemosa* (Papaveraceae)	12	rbcL	1428	*Meconopsis horridula_*JX087717.1 (Papaveraceae)	97	100	Genus
					*Meconopsis horridula*_ JX087712.1 (Papaveraceae)	97	99	
					*Meconopsis delavayi_*JX087688.1 (Papaveraceae)	97	99	
12A	*Macleaya microcarpa* (Papaveraceae)	12	rbcL	1428	*Macleaya microcarpa_*FJ626612.1 (Papaveraceae)	97	99	Family
					*Macleaya cordata_*U86629.1 (Papaveraceae)	97	99	
					*Coreanomecon hylomeconoides*_KT274030.1 (Papaveraceae)	100	98	
13A	*Hodgsonia macrocarpa* (Cucurbitaceae)	12	rbcL	1449	*Cucumis sativus* var. *hardwickii_*KT852702.1 (Cucurbitaceae)	100	98	Family
					*Cucumis sativus_*KX231330.1 (Cucurbitaceae)	100	98	
					*Cucumis sativus_*KX231329.1 (Cucurbitaceae)	100	98	
14A	*Malus yunnanensis* (Rosaceae)	12	rbcL	1428	*Cotoneaster franchetii_*KY419994.1 (Rosaceae)	100	99	Family
					*Vauquelinia californica_*KY419925.1 (Rosaceae)	100	99	
					*Cotoneaster horizontalis*_KY419917.1 (Rosaceae)	100	99	
					*Malus doumeri_*KX499861.1 (Rosaceae)	100	99	
15A	*Elaeagnus loureirii* (Elaeagnaceae)	12	rbcL	1428	*Elaeagnus macrophylla_*KP211788.1 (Elaeagnaceae)	100	99	Order
					*Elaeagnus* sp._KY420020.1 (Elaeagnaceae)	100	99	
					*Toricellia angulate*_KX648359.1 (Cornaceae)	99	99	
16A	*Rhododendron rex* subsp. *Fictolacteum* (Ericaceae)	12	rbcL	1428	*Rhododendron simsii*_GQ997829.1 (Ericaceae)	100	99	Family
					*Rhododendron ponticum*_KM360957.1 (Ericaceae)	98	99	
					*Epacris* sp._ L01915.2 (Ericaceae)	97	99	
17A	*Swertia bimaculata* (Gentianaceae)	12	rbcL	1443	*Swertia mussotii_*KU641021.1 (Gentianaceae)	98	99	Family
					*Gentianopsis ciliate_*KM360802.1 (Gentianaceae)	97	98	
					*Gentianella rapunculoides_*Y11862.1 (Gentianaceae)	97	99	
18A	*Primula sinopurpurea* (Primulaceae)	12	rbcL	1428	*Primula poissonii_*KX668176.1 (Primulaceae)	100	99	Genus
					*Primula chrysochlora*_KX668178.1 (Primulaceae)	100	99	
					*Primula poissonii*_KF753634.1 (Primulaceae)	100	99	
19A	*Paederia scandens* (Araceae)	12	rbcL	1443	*Pothos scandens_*AM905732.1 (Araceae)	96	99	Family
					*Pedicellarum paiei*_AM905733.1 (Araceae)	96	99	
					*Pothoidium lobbianum*_AM905734.1 (Araceae)	96	99	
20A	*Colocasia esculenta* (Araceae)	12	rbcL	1443	*Colocasia esculenta_*JN105690.1 (Araceae)	100	100	Species
					*Colocasia esculenta*_JN105689.1 (Araceae)	100	99	
					*Pinellia pedatisecta*_KT025709.1 (Araceae)	100	99	
21A	*Pholidota chinensis* (Orchidaceae)	12	rbcL		–	–	–	
22A	*Otochilus porrectus* (Orchidaceae)	12	rbcL		–	–	–	
23A	*Indosasa sinica* (Poaceae)	12	rbcL	1434	*Pleioblastus maculatus_*JX513424.1 (Poaceae)	100	100	Family
					*Oligostachyum shiuyingianum*_JX513423.1 (Poaceae)	100	100	
					*Indosasa sinica*_JX513422.1 (Poaceae)	100	100	
24A	*Camellia gymnogyna* (Theaceae)	12	rbcL	1428	*Camellia szechuanensis*_KY406778.1 (Theaceae)	100	100	Family
					*Pyrenaria menglaensis*_KY406747.1 (Theaceae)			
					*Camellia luteoflora*_KY626042.1 (Theaceae)			
25A	*Camellia sinensis* var. *assamica* (Theaceae)	12	rbcL	1428	*Camellia szechuanensis*_KY406778.1 (Theaceae)	100	100	Family
					*Pyrenaria menglaensis*_KY406747.1 (Theaceae)	100	100	
					*Camellia luteoflora*_KY626042.1 (Theaceae)	100	100	
					*Camellia sinensis* var. *assamica*_JQ975030.1 (Theaceae)	100	100	
26A	*Panicum incomtum* (Poaceae)	12	rbcL	1434	*Lecomtella madagascariensis_*HF543599.2 (Poaceae)	99	99	Family
					*Chasechloa madagascariensis*_KX663838.1 (Poaceae)	99	99	
					*Amphicarpum muhlenbergianum*_KU291489.1 (Poaceae)	99	99	
					*Panicum virgatum*_HQ731441.1 (Poaceae)	100	99

**Table 5:** BLAST results with extracted ITS sequence against GenBank

Query information					BLAST results		
Query_Sample ID	Query_Species (Family)	PCR cycles	Gene name	Length (bp)	Reference_Species (Family)	Query coverage	Identities
01A	*Manglietia fordiana* (Magnoliaceae)	12	ITS	369	*Magnolia virginiana_*DQ499097.1 (Magnoliaceae)	100%	95%
02A	*Manglietia fordiana* (Magnoliaceae)	12	ITS	349	*Magnolia virginiana_*DQ499097.1 (Magnoliaceae)	100%	95%
03A	*Schisandra henryi* (Schisandraceae)	12	ITS	676	*Schisandra pubescens_*AF263436.1 (Schisandraceae)	99%	100%
04A	*Schisandra henryi* (Schisandraceae)	12	ITS	676	*Schisandra pubescens_*JF978533.1 (Schisandraceae)	99%	99%
05A	*Phoebe neurantha* (Lauraceae)	12	ITS	518	*Phoebe neurantha_*FM957847.1 (Lauraceae)	100%	99%
06A	*Cinnamomum bodinieri* (Lauraceae)	12	ITS	603	*Cinnamomum micranthum* f. *kanehirae _*KP218515.1 (Lauraceae)	100%	99%
08A	*Holboellia latifolia* (Lardizabalaceae)	12	ITS	677	*Holboellia angustifolia* subsp. *angustifolia_*AY029790.1 (Lardizabalaceae)	100%	99%
09A	*Chloranthus erectus* (Chloranthaceae)	12	ITS	663	*Chloranthus erectus_*AF280410.1 (Chloranthaceae)	99%	99%
10A	*Sarcandra glabra* (Chloranthaceae)	12	ITS	667	*Sarcandra glabra_*KWNU91871 (Chloranthaceae)	100%	100%
11A	*Meconopsis racemosa* (Papaveraceae)	12	ITS	671	*Meconopsis racemosa_*JF411034.1 (Papaveraceae)	100%	99%
12A	*Macleaya microcarpa* (Papaveraceae)	12	ITS	612	*Macleaya cordata_*AY328307.1 (Papaveraceae)	99%	89%
13A	*Hodgsonia macrocarpa* (Cucurbitaceae)	12	ITS	614	*Hodgsonia heteroclita_*HE661302.1 (Cucurbitaceae)	100%	98%
14A	*Malus yunnanensis* (Rosaceae)	12	ITS	596	*Malus prattii_*JQ392445.1 (Rosaceae)	99%	99%
15A	*Elaeagnus loureirii* (Elaeagnaceae)	12	ITS	649	*Elaeagnus macrophylla_*JQ062495.1 (Elaeagnaceae)	99%	99%
16A	*Rhododendron rex* subsp. *fictolacteum* (Ericaceae)	12	ITS	646	*Rhododendron rex* subsp. *fictolacteum*_KM605995.1 (Ericaceae)	100%	100
17A	*Swertia bimaculata* (Gentianaceae)	12	ITS	626	*Swertia bimaculata _*JF978819.2 (Gentianaceae)	100	99%
18A	*Primula sinopurpurea* (Primulaceae)	12	ITS	631	*Primula melanops_*JF978004.1 (Primulaceae)	100%	99%
19A	*Paederia scandens* (Araceae)	12	ITS	–	*–*	–	–
20A	*Colocasia esculenta* (Araceae)	12	ITS	552	*Colocasia esculenta_*AY081000.1 (Araceae)	99%	99%
21A	*Pholidota chinensis* (Orchidaceae)	12	ITS	–	–	–	–
22A	*Otochilus porrectus* (Orchidaceae)	12	ITS	–	–	–	–
23A	*Indosasa sinica* (Poaceae)	12	ITS	604	*Oligostachyum sulcatum_*EU847131.1 (Poaceae)	98	99
24A	*Camellia gymnogyna* (Theaceae)	12	ITS	–	*–*	–	–
25A	*Camellia sinensis* var. *assamica* (Theaceae)	12	ITS	645	*Camellia sinensis* var. *sinensis*_FJ004871.1 (Theaceae)	99%	99%
26A	*Panicum incomtum* (Poaceae)	12	ITS	795	*Chasechloa egregia_*LT593967.1 (Poaceae)	100	98

**Fig. 2:**
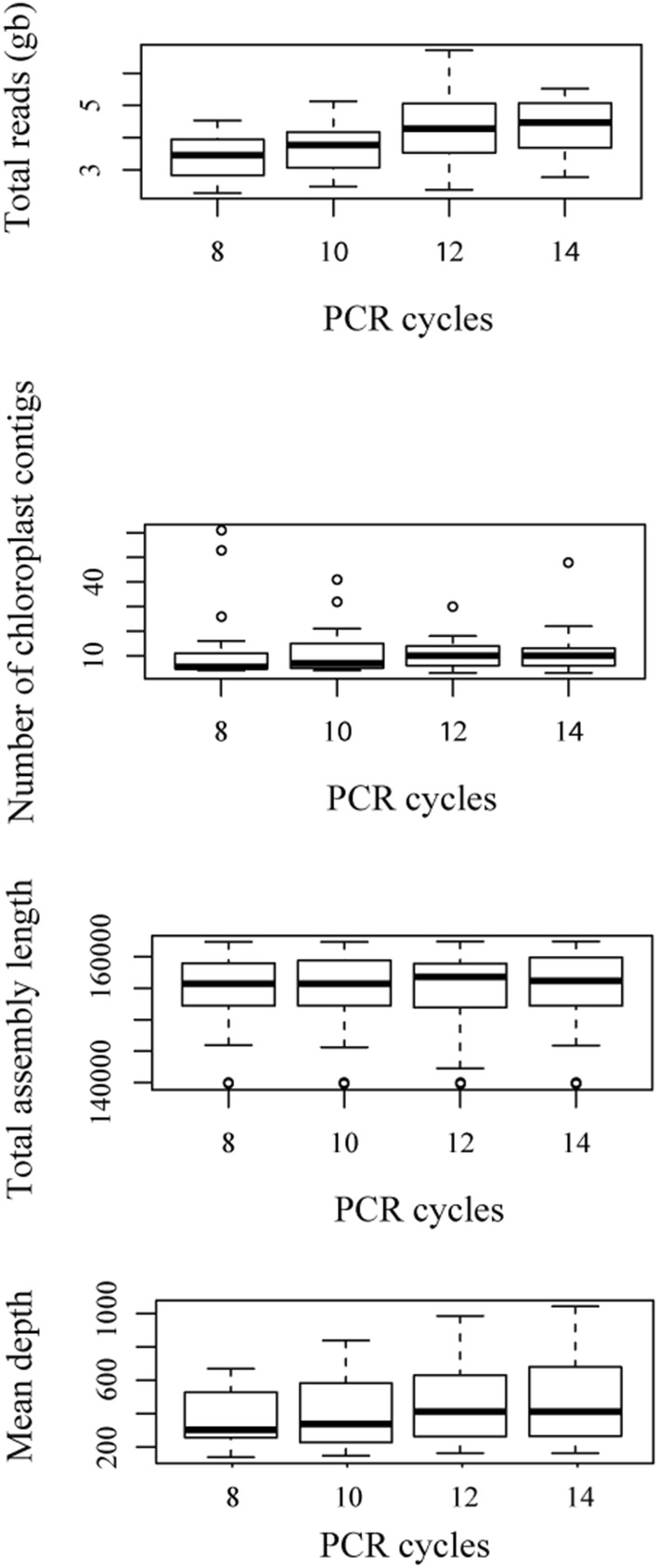
PCR cycles with raw data, contigs, and assembly length

One-way analyses of variance (ANOVA) were performed to test the total reads against PCR cycles, PCR cycles against plastid contig numbers, PCR cycles against plastid genome assembly length, PCR cycles against plastid mean-depth, and PCR cycles against plastid coverage. We found that was no significant correlation between PCR cycles and plastid contig numbers, PCR cycles and plastid genome assembly length, and PCR cycles and plastid coverage. There was, however, a significant positive correlation between the number of PCR cycles and the total number of reads, and PCR cycles and the plastid mean-depth (Fig. 2).

**Fig. 3:**
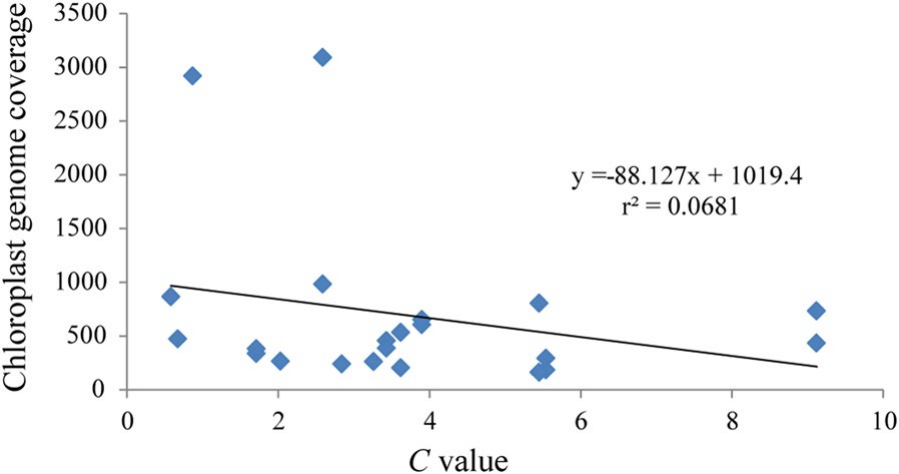
Plastome coverage versus C value (pg DNA per 1C) of all samples assembled in this study

Finally, when comparing plastome assembly coverage with C values of the species concerned we find a slight negative bit not significant correlation (Fig. 3), which would suggest, at least for our sampling, that plastome assembly coverage is not affected by nuclear genome size of the specimen concerned.

## Discussion

### Sequencing herbarium specimens from low amounts of starting DNA

Our current study successfully demonstrated the recovery of plastid genome sequences and rDNA sequences from herbarium specimens, some up to 80 years old. Our study used small amounts of starting tissue (c 1 cm^2^) and extremely low initial concentrations (500 pg) of degraded starting DNA. This success with a small amount of starting tissue is important, and demonstrates the practical feasibility of organelle genome and rDNA recovery with minimal impacts on specimens. These findings, in the context of studies by others (e.g. Bakker et al. [14]) confirm that genome skimming can be performed with limited sample destruction enabling relatively straightforward access to high-copy number DNA in preserved herbarium specimens spanning a wide phylogenetic coverage.

To accommodate the use of only 500 pg of input DNA, we modified the library protocol to remove the step of DNA fragmentation by sonication because the DNA was already highly degraded, we did not undertake any size selection, and we increased the number of PCR cycles to enrich the indexed library. After library preparation and Illumina paired-end sequencing, a sufficient number of read pairs (> 15,000,000) were generated for our 25 specimens and 100 libraries. This strategy allowed the generation of complete or near complete plastid genomes with depths ranging from 459 × to 2176 ×, and nuclear ribosomal units with a high sequencing depth (3 × to 567 ×) for 23 and 24 specimens respectively. Despite the low starting concentration, no plant or fungal contaminants were obviously detectable in the assembled plastomes and rDNA sequences.

For herbarium plastome assembly, the procedures and parameters for setting the sequence quality control, de novo assembly, blast search and genome annotation were followed as in Yang et al. [25]. The rate of our 25 specimens with 100 libraries was c. 5 h per specimen on a 3-TB RAM Linux workstation with 32 cores. It was not different significantly between fresh and herbarium specimens.

### Recovery of widely used loci in plant molecular systematics

A benefit of the genome skimming approach is that it can recover loci widely used in previous molecular systematics studies (e.g. Coissac et al. 2016 [12]). Here we recovered the standard *rbcL* DNA barcode region from 23/25 samples, the standard *matK* DNA barcode region from 23/25 specimens, the standard *trnH*-*psbA* DNA barcode region from 23/25 samples, the *trnL* intron from 23/25 samples, and the ITS1 and ITS2 from 20/25 to 19/25 samples respectively. In addition to the recovery of these standard DNA barcoding loci, we also recovered many other regions used as supplementary barcode markers (e.g. *atpF*-*H, psbK*-*I*). The data produced with this approach can thus contribute towards standard and extended DNA barcode reference libraries [12], in helping identify additional regions which are informative for any given clade [28], as well as producing data for phylogenomic investigations to elucidate the relationships amongst plant groups.

### Practical benefits

A primary motivation for this study was our own experiences with suboptimal DNA recovery from herbarium specimens using Sanger sequencing coupled with difficulty in accessing fresh material of some species. The success of this method using only small amounts of starting tissue from herbarium specimens is an important step to addressing these challenges. It makes sequencing type specimens a realistic proposition, which can further serves to integrate genetic data into the existing taxonomic framework. A second practical benefit is that field work is often not possible in some geographical regions where past collections have been made. Political instability and/or general inaccessibility can preclude current collecting activities, and where habitats have been highly degraded or destroyed, the species concerned may simply be no longer available for collection. Mining herbaria to obtain sequences from previously collected material can circumvent this problem. Thirdly, sequencing plastid genomes and rDNA arrays from specimens that are many decades old enables a baseline to be established for haplotype and ribotype diversity. This baseline can then be used to assess evidence for genetic diversity loss or change due to recent population declines or environmental change.

## Conclusions

This study confirms the practical and routine application of genome skimming for recovering sequences from plastid genomes and rDNA from small amounts of starting tissue from preserved herbarium specimens. The ongoing development of new sequencing technologies is creating a fundamental shift in the ease of recovery of nucleotide sequences enabling ‘new uses’ for the hundreds of millions of existing herbarium specimens [1, 10, 14, 16, 29]. This shift from Sanger sequencing to NGS approaches has now firmly moved herbarium specimens into the genomic era.
